# Malignant transformation of a long-standing submental dermoid cyst to a carcinosarcoma: a case report

**DOI:** 10.1186/s13256-016-1186-y

**Published:** 2017-01-11

**Authors:** Nadeena Sri Swarnagupta Jayasuriya, Samadarani Siriwardena, Wanninayake Mudiyanselage Tilakaratne, Suchithra Parthiepan

**Affiliations:** 1 Department of Oral and Maxillofacial Surgery, Faculty of Dental Sciences, University of Peradeniya, Peradeniya, Sri Lanka; 2 Department of Oral Pathology, Faculty of Dental Sciences, University of Peradeniya, Peradeniya, Sri Lanka; 3 Department of General Pathology, Teaching Hospital, Batticaloa, Sri Lanka

**Keywords:** Dermoid cyst, Submental, Malignant transformation, Carcinosarcoma, Case report

## Abstract

**Background:**

Submental dermoid cysts are uncommon midline cysts which occur due to entrapment of ectoderm between the second and third branchial arches during embryogenesis. Most dermoid cysts of the head and neck are benign, but rarely malignant transformation may occur. To the best of our knowledge, this is the first report of a carcinosarcoma arising in a submental dermoid cyst.

**Case presentation:**

A 42-year-old Sri Lankan Tamil man presented with a large cystic swelling in his submental region which was diagnosed as an extensive submental dermoid cyst. The cyst had been asymptomatic for 11 years but there was sudden enlargement and pain during the past 2 months. On surgical removal, a primary carcinosarcoma arising from part of the cyst wall was identified. After completion of radiotherapy, the disease was well controlled and he was disease free at 18 months.

**Conclusions:**

Although extremely rare, a dermoid cyst of the submental region can undergo malignant transformation. It can be successfully treated with surgical excision and radiotherapy.

## Background

Dermoid cysts are ectodermal inclusion cysts which arise in many parts of the body. Dermoid cysts are most frequently seen in the coccyx (44.5%) and ovaries (42.1%), followed by the head and neck [1]. After analyzing1495 dermoid cysts, New and Erich reported a prevalence of 6.94% in the head and neck region [2]. Dermoid cysts of the floor of the mouth (DCFM) account for 1.6 to 6.5% of all body dermoid cysts [3]. According to Meyer, a true dermoid cyst contains both epithelial and connective tissue components; it is thus a compound cyst [4].

Clinically, dermoid cysts expand slowly over many years. Although congenital, DCFM may occur at any age after birth (average 15 to 35 years). Gender distribution is almost equal [4]. DCFM are classified according to their anatomical site (median and lateral), the relationship to the mylohyoid muscle (sublingual and submental), and by the germ cell layers (epidermoid, dermoid, and teratoid) [4]. DCFM can cause many complications due to the anatomical position and the size of cysts. Apart from dysphagia, difficulty in chewing, and altered speech, intubation during general anesthesia can also be a significant concern. The treatment of choice for a DCFM is surgery. Meticulous excision of the cyst including all tracts and adhesions is advised for a complete cure [5]. Malignant transformation in a dermoid cyst is rare and usually unexpected. This is the first report of a malignant transformation of a submental dermoid cyst into a carcinosarcoma.

## Case presentation

A 42-year-old Sri Lankan Tamil man presented with a large cystic swelling in his submental region. There were no obvious intra-oral extensions. The cyst had enlarged gradually over 11 years but there was rapid enlargement over the last 2 months. Apart from discomfort and pain, which was of recent onset, he was asymptomatic.

A computed tomography (CT) scan showed a well-demarcated cyst in his submental region measuring 4×5.4×6 cm. A solid mass of 1.4×2.9 cm was also noted at the medial margin of the cyst wall and appeared suspicious. The adjacent soft tissues and his lymph nodes were uninvolved.

**Fig. 1:**
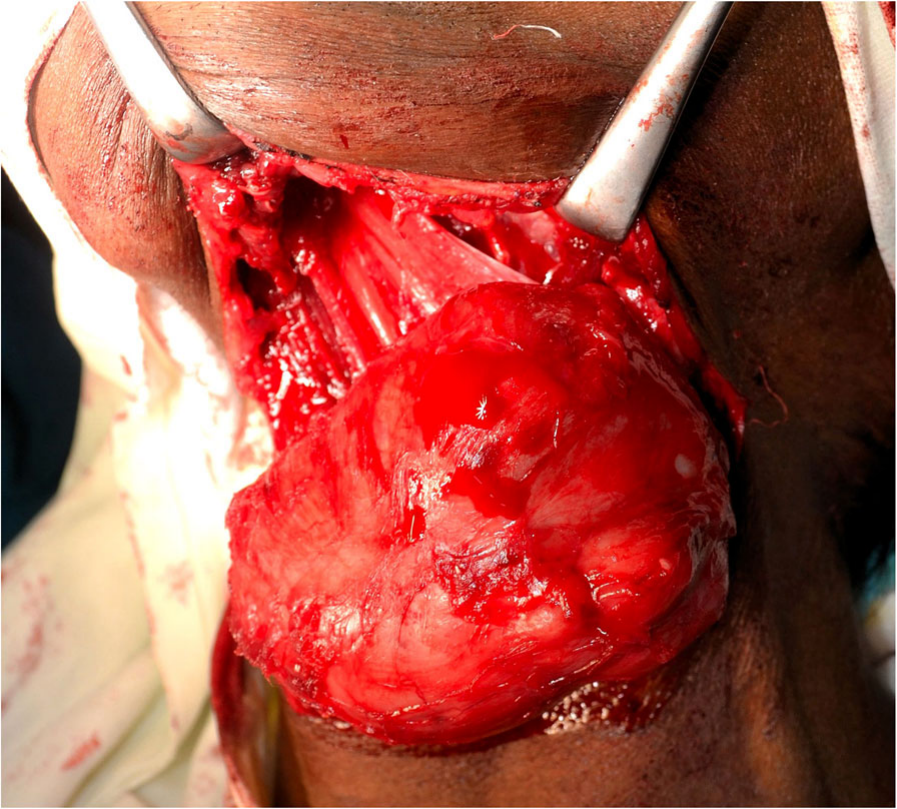
Intraoperative photograph showing the large dermoid cyst being removed through a submental incision

**Fig. 2:**
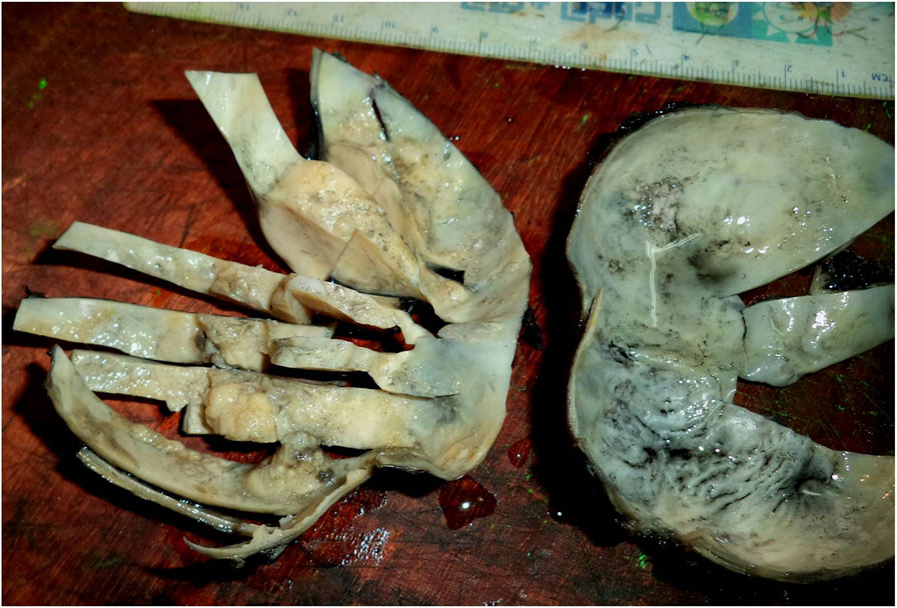
Macroscopic view of the cyst after formalin fixation. The malignant transformation is seen at the inner wall of the dermoid cyst

**Fig. 3:**
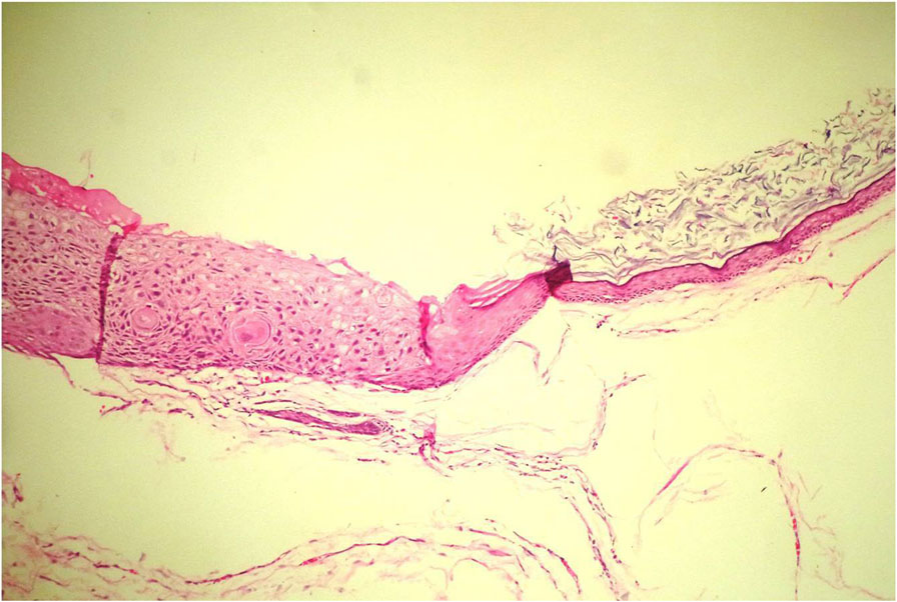
An area of malignant transformation (×40). Typical orthokeratinized epithelial lining of dermoid cyst and severely dysplastic epithelium

**Fig. 4:**
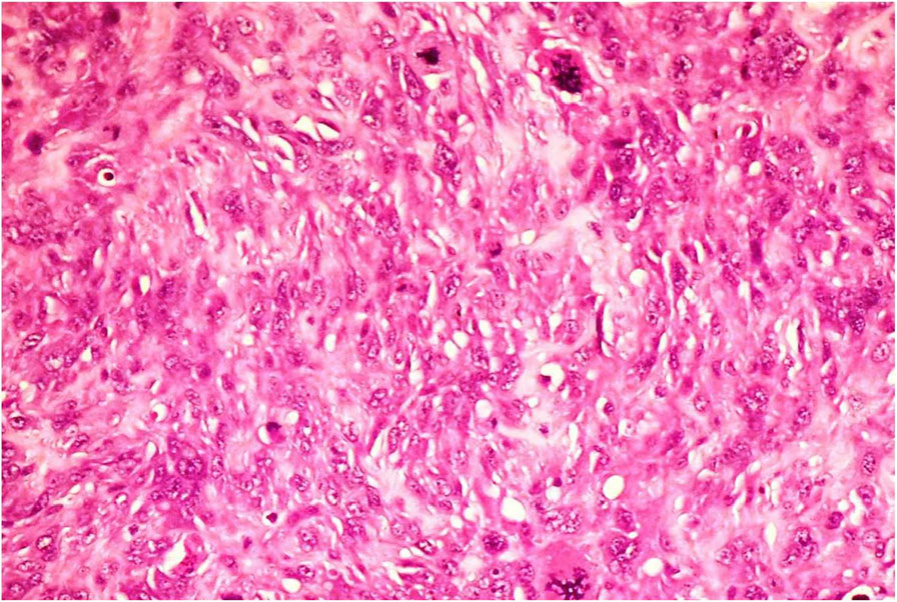
Hematoxylin and eosin stained section (×400) demonstrates a sarcomatous region of the cyst wall with cellular atypia and mitoses

The lesion was surgically removed under general anesthesia using a submental approach (Fig. 1). On macroscopic examination, the lesion was well encapsulated with no obvious extracapsular invasion. The cut surface showed a large cyst with a growth into the lumen (Fig. 2). Histopathology showed a characteristic dermoid cyst epithelial lining; regular orthokeratinized stratified squamous epithelium toward one area. It was interesting to note that there was gradual change of the dermoid cystic squamous epithelium from moderate to severe epithelial dysplasia which then transformed into an invading malignancy (Fig. 3). Toward the growth, marked cytological atypia with numerous mitoses were observed. An immunohistochemical stain for cytokeratin (AE1/AE3) was positive only in the carcinomatous areas. The central sarcomatous area of the lesion was negative for immunohistochemical markers S-100 protein, smooth muscle actin, desmin, human melanoma black 45 (HMB-45), and leukocyte common antigen (LCA). A diagnosis of carcinosarcoma arising from a dermoid cyst was confirmed (Fig. 4).

The lesion was completely excised by surgery. Radiotherapy was started 4 weeks and 3 days after surgery, following complete healing of the surgical wounds. An equal dose of radiotherapy was administered to the primary site and bilateral neck to include level I to III (60 Grays, five fractions/week, leading to 6 weeks of treatment). Concomitant chemotherapy was not administered considering the microscopic nature of the primary disease, absence of distant metastasis in CT scan (neck and lungs), and potential side effects. Radiation-induced mucositis and dry mouth were the most significant complications of radiotherapy and were managed conservatively. He was free of recurrences at review at 18 months.

## Discussion

Malignant transformation in a dermoid cyst is rare (less than 2 %) and is usually seen in ovaries and testes [6]. Squamous cell carcinoma is the most common malignancy diagnosed in dermoid cysts followed by adenocarcinoma [7]. Intracranial and lumbar dermoid cysts are examples in which squamous cell carcinoma had arisen within the cyst walls [8, 9]. The first case of malignant transformation in a DCFM was reported by Devine and Jones in 2000 [10]. They described a squamous cell carcinoma which occurred in a sublingual dermoid cyst in a 56-year-old man. Subsequently, a second case report in 2010 published the presence of dual malignancies (simultaneous squamous cell carcinoma and osteosarcoma) in the wall of a sublingual dermoid cyst [6]. Similarly, the submental dermoid cyst in our patient developed into a carcinosarcoma; it showed malignant transformation in both the epithelial and the mesenchymal tissues. To the best of our knowledge, this is the first report of a mixed malignancy arising in a submental dermoid cyst. Although multiple malignancies which arise simultaneously within cyst walls are rare, they are documented more frequently in the ovaries.

The pathogenesis of a carcinosarcoma is explained by multiclonal or monoclonal hypotheses. The multiclonal hypothesis describes the origin of the two forms of malignancies from separate epithelial and mesenchymal cell lines [7]. In contrast, the monoclonal hypothesis proposes the origin of a carcinoma and a sarcoma from a common totipotential stem cell [11]. The use of molecular markers helps in the differentiation of the above.

The exact cause for malignant transformation within the cyst wall is uncertain. A diameter more than 10 cm, age more than 45 years, rapid growth, thickening of the cyst wall, and adherence to an adjacent structure are considered risk factors for malignant transformation in dermoid cysts and teratomas in the ovaries [6, 12]. In addition, the tumor size, the spread of the disease, and the patient’s age are thought to influence survival after treatment. Due to its rarity, the prognosis of DCFM is not known. Apart from the long duration of 11 years, this patient did not have any other risk factor for malignant transformation.

## Conclusions

Malignant transformation was clinically suspected due to the sudden increase in size and associated pain. Therefore, we recommend that clinicians be vigilant of malignant change in cases of rapid enlargement of a dermoid cyst. Surgery and radiotherapy helped to control the disease and our patient is disease free at 18 months. Due to the rare nature of the disease, a treatment protocol cannot be recommended. Furthermore, a long-term follow up of at least 5 years is required to comment on the success of treatment.

## Abbreviations

CT: Computed tomography
DCFM: Dermoid cysts of the floor of the mouth
HMB-45: Human melanoma black 45
LCA: Leukocyte common antigen
